# Survey of Massachusetts peer recovery coaches’ attitudes toward the use of psychedelics to treat substance use disorders

**DOI:** 10.1186/s13722-024-00517-y

**Published:** 2024-11-25

**Authors:** Veronica Szpak, Amanda Kim, Zachary Sager, Joji Suzuki

**Affiliations:** 1 Department of Psychiatry, Brigham and Women’s Hospital, 60 Fenwood Rd, Boston, MA 02115 USA; 2 Harvard Medical School, Boston, MA USA; 3 Dana-Farber Cancer Institute, Boston, MA USA

**Keywords:** Peer recovery coach, Psychedelics, Psychedelic-assisted therapy, 12-steps, Survey

## Abstract

**Background:**

There has been a growing interest in the use of psychedelics for therapeutic purposes. However, there is a lack of research on peer recovery coaches' attitudes toward the use of psychedelics for SUD treatment. Therefore, we conducted a survey of peer recovery coaches in Massachusetts to gain insight into their attitudes toward the use of psychedelics to treat SUDs.

**Methods:**

Peer recovery coaches in Massachusetts were invited to participate in an online survey between August and October 2023. The survey collected respondents’ demographics, socioeconomic characteristics, personal substance use history, opinions on psychedelics for addiction treatment, and spiritual experiences.

**Results:**

146 individuals completed the survey. The mean age was 48.7 years (SD 11.2), 61% identified as female, 74% were employed as peer recovery coaches, and 43% were Certified Addiction Recovery Coaches (CARC). 70.7% reported utilizing 12-step programs, and 76% reported having a personal history of using psychedelics. The majority of participants agreed that they would feel comfortable being a coach for someone using psychedelics to treat SUDs. However, a significant number of participants expressed concerns. Those who had utilized 12-steps were more likely to express concerns about the dangers of using psychedelics to treat SUD. Conversely, participants with a personal history of psychedelic use were more likely to support the use of psychedelics for the treatment of SUDs.

**Conclusions:**

While peer recovery coaches express support for using psychedelics to treat SUD, they also voice concerns about the potential risks.

## Background

There has been a resurgence of research focused on the therapeutic application of psychedelic drugs [1]. Studies conducted with psychedelics prior to the 1970s suggested a therapeutic potential for the treatment of substance use disorders (SUDs) but exhibited numerous methodological shortcomings [2, 3]. A meta-analysis examined the efficacy of lysergic acid diethylamide (LSD) in the treatment of alcohol use disorder in six trials and found evidence of LSD reducing alcohol use [4]. Psilocybin and ketamine have been researched for pilot double-blind randomized trials for SUDs demonstrating promising results [5–8]. Additionally, a systematic review examined seven trials and found that ketamine was associated with abstinence across multiple substances [9]. As the perception of utilizing psychedelics as potential treatments shifts, surveys of mental health and addiction treatment providers have generally demonstrated broad support for the therapeutic applications of psychedelics for treating mental health conditions and SUDs [10, 11].

Peer recovery coaches are increasingly an impactful and rapidly growing group of professionals in healthcare settings who support individuals in recovery from SUD [12, 13]. Additionally, peer recovery coaches are individuals with lived experience of successful recovery who obtain training to become certified coaches to foster hope, provide social support, and respect all pathways to recovery. The growing body of evidence points to their role in improving SUD-related outcomes [14, 15]. Historically, 12-step programs such as Alcoholics Anonymous (AA) or Narcotics Anonymous (NA), which are also composed of peers in recovery, have been abstinence-based. Although 12-step programs have more recently shown greater receptiveness to the use of medications for recovery, research has not investigated the attitudes of peer recovery coaches regarding the use of psychedelics to treat SUDs. To address this gap, we conducted a survey of peer recovery coaches in Massachusetts to better understand their attitudes toward the use of psychedelics to treat SUDs.

## Methods

### Setting

The study was approved by the Mass General Brigham (MGB) IRB and conducted at Brigham and Women’s Hospital in Boston, Massachusetts.

### Participants and recruitment

Inclusion criteria consisted of adults 18 years of age or older who had completed peer recovery coach training in Massachusetts. Respondents were recruited anonymously by email which contained a link to the survey. Emails indicated the survey was seeking their opinions regarding psychedelic-assisted treatments for SUD. Recruitment emails were distributed by the Department of Public Health of the State of Massachusetts and private training agencies on behalf of the study staff and sent to all individuals who had ever completed the recovery coach training in the state. The survey invitations were sent between August and October of 2023.

### Survey

The survey was created and administered using the MGB REDCap. The survey collected the respondents’ demographics, personal history of addiction and psychedelic use, opinions regarding psychedelics for addiction treatment, and possible connections between their personal psychedelic experiences with the spiritual awakenings as outlined in the principles of 12-step (AA/NA). Response choices consisted of a 5-point Likert scale, from *strongly agree to strongly disagree.* A recovery coach provided input on the development of the survey, as individuals with lived experience are becoming increasingly involved in research designs and interventions [16, 17].

### Analytic strategy

All statistical analyses were conducted in R Studio version 4.2.2 [18]**.** The data was summarized using descriptive statistics. The proportion of respondents agreeing and disagreeing with each statement was calculated by collapsing responses with "agree" and "strongly agree”, and “disagree” and “strongly disagree”. Chi-square tests compared categorical variables (agree vs disagree) between those who do and do not utilize AA/NA 12-steps and those with and without a personal history of psychedelic use. Contingency tables were created for the variables and the chi-square tests were conducted using the chisq.test() function. Alpha was set at 0.05 for all analyses.

## Results

Invitations were sent to 1,085 individuals, and 146 valid responses were obtained, with a response rate of 13.5%. Participants had a mean age of 48.7 years (SD 11.2), 61% identified as female, 74% were employed as peer recovery coaches, and 43% were Certified Addiction Recovery Coaches (CARC). 70.7% reported utilizing AA/NA 12 steps and 76% reported a personal history of psychedelic use. Additional demographic data are presented in Table 1.

**Table 1:** Demographic and clinical characteristics of respondents

Participant characteristics	Total *n* = 146
Age (SD)	48.74 (11.2)
Missing Responses	2
Gender, n (%)	
Male	52 (35.6%)
Female	89 (61%)
Non-Binary	3 (2%)
Missing Responses	2 (1.4%)
Education, n (%)	
High school diploma/GED/Some high school	70 (47.9%)
Associates degree	21 (14.4%)
Bachelor’s degree	38 (26%)
Graduate degree	14 (9.6%)
Missing Responses	3 (2.1%)
Religion, n (%)	
Catholic	24 (16.4%)
Protestant	8 (5.5%)
Baptist	3 (2.1%)
Jewish	4 (2.7%)
Other	16 (11%)
Spiritual not religious	63 (43.1%)
Atheist or Agnostic	7 (4.8%)
Prefer not to say	21 (14.4%)
CARC Certified, n (%)	
Yes	64 (43.8%)
No	76 (52.1%)
Missing Responses	6 (4.1%)
Currently working as a Recovery Coach, n (%)	
Yes	108 (74%)
No	37 (25.3%)
Missing Responses	1 (0.7%)
In Recovery, n (%)	
Yes	140 (95.9%)
No	5 (3.4%)
Missing Responses	1 (0.7%)
Psychedelic Use, n (%)	
Classic Psychedelics (LSD, psilocybin, mescaline, etc.)	99 (68.3%)
Dissociatives (Ketamine, PCP)	39 (26.7%)
Novel Psychedelics (NBOMe, 2C-I, etc.)	8 (5.5%)
Empathogens (MDMA or ecstasy)	59 (40.4%)
Other	8 (5.5%)
None	34 (23.3%)
Missing Response	1 (0.7%)
Recovery and Substance use history	n = 140
Recovery Support Engagement, n (%)	
AA/NA 12 Steps	99 (70.7%)
SMART Recovery	36 (25.7%)
Dharma Recovery	31 (22.1%)
Faith Based Approaches	30 (21.4%)
Mindfulness	91 (65%)
Other	49 (35%)
History of Substances, n (%)	
Opioids	94 (67.1%)
Alcohol	115 (82.1%)
Cannabis	85 (60.7%)
Cocaine	89 (63.5%)
Amphetamines	49 (35%)
Other	22(15.7%)

140 answered “yes” to the question “Do you identify as a person in recovery?”

Most respondents (67.8%) reported being knowledgeable about psychedelics and comfortable being a coach for someone using psychedelics to treat SUD (67.8%). The majority agreed that coaches should receive training on how psychedelics might help treat SUD (84.3%) but that more research is needed before making them available for the treatment of SUD (69.9%). A minority agreed that psychedelics are harmful for treating SUD (15.8%), that psychedelics could make relapse more likely (28.8%), and that one cannot be in recovery if using psychedelics for any reason (12.3%). In contrast, only a minority supported making psychedelics legally available now for the treatment of SUD (42.5%) or for recreational purposes (24.0%), and a substantial proportion endorsed having concerns about the potential dangers of psychedelics (57.5%).

Respondents utilizing 12-steps were more likely to have concerns about the dangers of using psychedelics to treat SUD (68.7% vs 36.2%, p < 0.001) and felt that 12-step members would not support the use of psychedelics for the treatment of SUD (78.8% vs 68.1%, p = 0.02). Conversely, respondents with a personal history of psychedelic use were more likely to support the use of psychedelics for the treatment of SUDs (48.5% vs 23.5%, p < 0.01). Results comparing responses between those who do and do not utilize 12-steps, as well as between those who do and do not have personal experience with psychedelics are summarized in Table 2.

**Table 2:** Survey questions and responses

Opinions on Psychedelics Agree, Neither, or Disagree	Total (n = 146) n, % of Agree	AA/NA 12 Steps (n = 99) n, % of Agree	Non-AA/NA 12 Steps (n = 47) n, % of Agree	Chi-Square p-value	Psychedelic History (n = 111) n, % of Agree	No Psychedelic History (n = 34) n, % of Agree	Chi-Square p-value
I am knowledgeable about psychedelics	99 (67.8%)	63 (63.6%)	36 (76.6%)	0.21	84 (75.7%)	15 (44.1.3%)	< 0.0001
Psychedelics should be made legally available for treating SUD	62 (42.5%)	39 (39.4%)	23 (49%)	0.15	54 (48.6%)	8 (23.5%)	< 0.01
I would be comfortable being the coach with a patient who is using psychedelics for treating SUD	99 (67.8%)	62 (62.6%)	37 (78.7%)	0.09	79 (71.2%)	20 (58.8%)	0.38
Psychedelics should be made legal for recreational purposes	35 (24%)	22 (22.2%)	13 (27.7%)	0.78	29 (26.1%)	6 (17.6%)	0.5
I have concerns about the dangers of using psychedelics for treating SUD	85 (57.5%)	68 (68.7%)	17 (36.2%)	< 0.001	68 (61.3%)	16 (47.1%)	< 0.01
Psychedelics are harmful for treating SUD	23 (15.8%)	17 (17.2%)	6 (12.8%)	0.02	18 (16.2%)	5(14.7%)	0.11
Using psychedelics for SUD could make relapse more likely	42 (28.8%)	33 (33.3%)	9 (19.1%)	0.03	33 (29.7%)	9 (26.5%)	0.13
You cannot be in recovery if you are using psychedelics for any reason	18(12.3%)	15 (15.2%)	3 (6.4%)	0.12	12 (10.8%)	6 (17.6%)	0.32
Some psychedelics are addictive	63 (43.2%)	45 (45.5%)	18 (38.3%)	0.54	53(47.7%)	9 (26.5%)	0.02
Recovery coaches should receive training on how psychedelics might be used to treat SUD	119 (84.3%)	79 (87.8%)	40 (85.1%)	0.71	94 (84.7%)	24 (70.6%)	0.13
More research is needed before psychedelics are made available for treating SUD	102 (69.9%)	71 (71.2%)	31 (66%)	0.23	77(69.4%)	24 (70.6%)	0.15
Overall, people in recovery would not support using psychedelics for treating SUD	54 (37%)	41 (41.4%)	13 (27.7%)	0.16	40 (36%)	14 (41.2%)	0.03
Overall, 12-step members and sponsors would not support using psychedelics for treating SUD	110 (75.3%)	78 (78.8%)	32 (68.1%)	0.02	88(79.3%)	21 (61.7%)	0.03
Psychedelics can allow a person to have a spiritual experience	82 (56.2%)	55 (55.5%)	27 (57.4%)	0.9	71(64%)	11 (32.4%)	< 0.01
Going through the 12-steps can allow for a person to have a spiritual awakening	116 (79.5%)	92 (92.9%)	24 (51.1%)	< 0.0001	96 (86.5%)	19 (55.9%)	< 0.001
A spiritual awakening from 12-steps and a spiritual experience from psychedelics are the same thing	18 (12.3%)	13 (13.1%)	5 (10.6%)	0.06	17 (15.3%)	1 (2.9%)	0.02

## Discussion

As interest in the applications of psychedelics for the treatment of SUD gains momentum, the attitudes of peer recovery coaches have received little attention. Even though respondents to this survey with a personal history of psychedelic use expressed more supportive views, results suggest that this sample of peer recovery coaches are concerned about the potential harms, the addictive potential of psychedelics, and remain skeptical that 12-step members would be supportive. These findings are consistent with the literature on 12-step programs and its model of an abstinence-focused approach to recovery, as well as with prior reports that suggest personal history has a strong influence on current attitudes and beliefs [19, 20]. That most (76%) of the responding peer recovery coaches reported prior experience with psychedelics is notable, and further research is warranted to determine whether this prevalence is representative of peer recovery coaches in general.

Nevertheless, most respondents felt that peer recovery coaches should receive training on how psychedelics may be used to treat SUDs and agreed that they would feel comfortable coaching a patient who is using psychedelics for SUD treatment. This is aligned with the core mission of peer recovery coaches in supporting patients with different paths to recovery, while also suggesting that additional education is necessary to support coaches in meeting this goal. Taken together, these findings highlight how the views of peer recovery coaches may have implications for how psychedelic treatments are implemented.

There are several limitations to this study. The response rate was low, and our sample was not representative. Respondents were individuals undergoing training in Massachusetts, limiting our ability to generalize to peer recovery coaches more broadly. Our sample was also homogenous in terms of sociodemographic characteristics. In addition, the survey did not ask about work settings. The survey asked about opinions on psychedelics in general and did not differentiate between particular drugs, potentially obscuring nuances around perceptions of benefits and harms pertaining to individual agents. Finally, 76% of our sample used psychedelics, and strong beliefs either for or against psychedelic use may have compelled individuals to complete the survey, biasing results.

## Conclusions

While still expressing support, this sample of peer recovery coaches appears to harbor concerns about the potential harms of psychedelics. Given their growing role in addiction treatment, further research is warranted to better understand the attitudes toward the use of psychedelics for treating SUDs among peer recovery coaches.

## Data Availability

The datasets used and/or analyzed during the current study are available from the corresponding author on reasonable request.
